# Acceptance of generative AI–assisted medical decision-making among Chinese physicians and patients and its ethical determinants: a cross-sectional survey

**DOI:** 10.3389/fpubh.2026.1833489

**Published:** 2026-08-05

**Authors:** Zhen Qian, Xizheng Li, Yingxue Li, Lina Wang, Qian Xu, Feng Cao, Yuwen Lyu, Junrong Liu

**Affiliations:** 1 Institute of Humanities and Social Sciences, Guangzhou Medical University, Guangzhou, Guangdong, China; 2 School of Health Management, Guangzhou Medical University, Guangzhou, Guangdong, China; 3 Division of Nephrology, Nanfang Hospital, Southern Medical University, Guangzhou, Guangdong, China; 4 Medical Ethics Office, The Affiliated Guangdong Second Provincial General Hospital of Jinan University, Guangzhou, Guangdong, China; 5 School of Medical Humanities, China Medical University, Shenyang, Liaoning, China; 6 School of Marxism, Guangzhou Medical University, Guangzhou, Guangdong, China

**Keywords:** acceptance intention, ethical governance, generative artificial intelligence, healthcare AI, medical decision-making, physician–patient perspectives

## Abstract

**Objective:**

Generative artificial intelligence (GAI) is increasingly being integrated into medical decision-making, yet ethical challenges continue to hinder its widespread clinical implementation. Existing research has paid limited attention to the ethical determinants of acceptance and differences in perceptions among key stakeholder groups. This study examined acceptance of GAI-assisted medical decision-making among Chinese physicians, patients, and other healthcare stakeholders, identified its ethical determinants, and explored differences across stakeholder groups to inform ethical governance.

**Methods:**

A cross-sectional survey was conducted using a multistage sampling strategy across healthcare institutions in Henan and Guangdong provinces, China, between December 2025 and January 2026. A total of 533 participants, including healthcare professionals, patients, and other stakeholders, were recruited. A self-developed four-dimensional scale assessing perceived functional value, perceived ethical risk, acceptance intention, and ethical governance expectations was developed through literature review, expert consultation, and pilot testing. Multivariable linear regression analysis was performed using SPSS version 26.0 to identify factors associated with acceptance intention.

**Results:**

Participants reported a moderate level of acceptance of GAI-assisted medical decision-making (3.68 ± 0.70), while ethical governance expectations received the highest mean score (4.03 ± 0.76). Multivariable regression analysis showed that perceived functional value (*β* = 0.528, *p* < 0.001) and ethical governance expectations (β = 0.392, p < 0.001) were significant positive predictors of acceptance intention, whereas perceived ethical risk showed a statistically significant but relatively weak negative association. Healthcare professionals reported higher perceived ethical risk than patients (3.31 ± 0.75 vs. 3.23 ± 0.91, *p* < 0.05). Participants with postgraduate education reported the strongest ethical governance expectations (4.33 ± 0.59), whereas patients with chronic diseases perceived lower data privacy risk than those without chronic diseases (2.71 ± 1.08 vs. 3.57 ± 0.99, *p* < 0.001).

**Conclusion:**

Acceptance of GAI-assisted medical decision-making among Chinese stakeholders is primarily driven by perceived functional value and ethical governance expectations, with significant differences in ethical perceptions across stakeholder groups. These findings provide empirical evidence for developing targeted ethical governance frameworks to facilitate responsible GAI integration into clinical practice.

## 1 Introduction

Generative artificial intelligence (GAI), represented by large language models (LLMs), has increasingly penetrated various domains of healthcare since the introduction of ChatGPT. With capabilities such as natural language interaction, multimodal data processing, and clinical knowledge generation, GAI has been applied in multiple stages of medical decision-making, including diagnostic assistance, treatment recommendation, and support for physician–patient communication (1–4). As one of the key technologies driving the digital transformation of healthcare, GAI has the potential to improve the efficiency of clinical decision-making and optimize the allocation of healthcare resources. It also provides technological support for the implementation of precision medicine and personalized healthcare services. Consequently, the application of GAI in medical decision-making has become an important topic in interdisciplinary research (5). Nevertheless, existing studies suggest that the large-scale implementation of GAI in healthcare depends not only on technological advancement but also on the interaction among technical performance, ethical governance, and the perceptions of multiple stakeholders (6).

Extensive cross-disciplinary research spanning human–computer interaction and consumer behavior has constructed mature analytical frameworks for public acceptance of intelligent systems (7–9), systematically validating core constructs including trust in artificial intelligence, algorithm aversion, perceived value, and perceived risk. These works lay important theoretical foundations for analyzing users’ adoption attitudes toward emerging AI tools and have been widely replicated across multiple application scenarios. Meanwhile, a growing body of clinical GAI studies published between 2022 and 2026 has converged on two broadly consistent conclusions: generative AI delivers clear efficiency gains in clinical auxiliary work, and universal ethical constraints including data privacy risks and algorithm bias shape users’ willingness to adopt such tools (10). These consistent findings form a valuable foundational consensus for follow-up research and have been supported by evidence from multiple national contexts.

Despite these advances, three key underexplored directions remain in the existing literature. First, most cross-disciplinary acceptance theories are generalized without targeted verification under the unique ethical constraints and doctor–patient relational norms of clinical medical scenarios. Second, existing quantitative studies mostly recruit either physicians or patients as isolated samples, while synchronous comparative data of the two core medical stakeholders remain scarce (11), especially quantitative evidence collected within China’s differentiated medical resource allocation and collectivist cultural context (12, 13). Third, although prior ethical discussions have repeatedly addressed regulatory and supervision needs, few quantitative studies incorporate ethical governance expectations as an independent predictive variable into classic technology acceptance models, leaving the linkage between regulatory demand and AI adoption intention insufficiently verified. These gaps provide the rationale for the cross-sectional design adopted in this study.

Against this background, the present study focuses on healthcare professionals (with physicians as the core subgroup) and patients in China as the primary research population, while also including other relevant stakeholder groups as reference participants. Using a cross-sectional survey combined with multivariable linear regression analysis, this study was designed to address the gaps identified above. Specifically, the study pursues three main objectives: (1) to systematically assess the level of acceptance of GAI-assisted medical decision-making among Chinese physicians and patients; (2) to quantitatively examine the driving effects of ethical factors—including perceived ethical risk and ethical governance expectations—on acceptance intention and to clarify their relative importance compared with perceived functional value; and (3) to analyze cognitive differences across demographic and clinical subgroups.

To clarify the supplementary value of this work relative to existing literature, three modest incremental advances are summarized as follows. First, empirically, unlike most prior surveys focusing on a single medical stakeholder, this study collects matched data from both healthcare professionals and patients synchronously, offering an additional comparative perspective on inter-group cognitive heterogeneity. Second, theoretically, on the basis of the mature Technology Acceptance Model, this study takes ethical governance expectations as an extended variable for quantitative verification, providing a minor expansion to existing analytical frameworks for medical AI acceptance. Third, contextually, this paper delivers cross-sectional quantitative data sampled from two Chinese provinces, adding localized empirical supplements to global research that is currently dominated by evidence from Western healthcare systems. Each of the three underexplored research angles identified from prior literature corresponds directly to one of the above core designs, clarifying the targeted positioning of this study within global generative AI acceptance research.

On the basis of the above research positioning, this study further clarifies the theoretical foundation and variable relationships of the analytical framework, so as to form a conceptual bridge between the research questions and the subsequent empirical analysis. This study constructs an extended analytical framework anchored in the classic Technology Acceptance Model (TAM) (7), integrated with risk perception theory (14) and relevant ethical governance perspectives (12, 15). The original TAM proposes that users’ acceptance of new technologies is primarily determined by perceived usefulness and perceived ease of use, and this core logic has been widely validated in the field of healthcare technology adoption. Given the prominent ethical attributes of generative AI–assisted medical decision-making, this study retains perceived functional value (corresponding to perceived usefulness in TAM) and acceptance intention as core constructs, and introduces two context-specific extended variables: perceived ethical risk and ethical governance expectations. Perceived ease of use is not included as a core variable, as the operation of AI-assisted decision-making is primarily undertaken by healthcare professionals rather than general users, and the core focus of this study lies in ethical attributes rather than operational usability.

Specifically, perceived functional value reflects the practical utility of generative AI in improving diagnostic efficiency and optimizing clinical decision-making. Anchored in the perceived usefulness dimension of TAM, it has been consistently validated as a key positive predictor of technology acceptance; in the medical GAI scenario, higher perceived functional value means users believe AI can effectively improve diagnostic accuracy and work efficiency, and is thus expected to be positively associated with acceptance intention. Ethical risk refers to potential ethical concerns such as privacy leakage, algorithmic bias and diminished physician autonomy in the application of generative AI. Drawing on risk perception theory, when users perceive higher levels of ethical risks, their sense of uncertainty about the technology increases, which in turn reduces their acceptance intention (16). Accordingly, ethical risk is predicted to exert a negative effect on acceptance. Ethical governance expectations represent the public’s demand for normative supervision of medical AI applications. Integrating perspectives from AI ethical governance research, we argue that public ethical governance expectations are not equivalent to resistance to technology; instead, higher expectations for standardized supervision reflect users’ recognition of the benign development of AI, which will further enhance their confidence in and acceptance of AI-assisted medical decision-making. Thus, ethical governance expectations are hypothesized to positively predict acceptance intention (17).

On the basis of the above logical deductions, this study proposes three corresponding research hypotheses. To empirically test the above theoretical framework and variable relationships, this study adopts a cross-sectional survey design. The specific study design, measurement instruments and analytical procedures are presented in the following section.

## 2 Materials and methods

### 2.1 Study design and ethical approval

This study employed a cross-sectional survey with a multistage field survey design. The empirical investigation was conducted between December 2025 and January 2026 across multiple healthcare institutions in Henan and Guangdong provinces, China. Focusing primarily on physicians and patients, the study aimed to examine acceptance of generative artificial intelligence (GAI)–assisted medical decision-making and its ethical determinants among physicians, patients, and other healthcare stakeholders in China.

The study protocol was approved by the institutional medical ethics committee (Approval No. 202601016). All procedures were conducted in accordance with the ethical principles of the Declaration of Helsinki. Participation was voluntary, and informed consent was obtained from all respondents. The survey was conducted anonymously to ensure confidentiality.

### 2.2 Sampling strategy

This study adopted a multistage field survey design, and the survey was conducted in Guangdong and Henan Province, China. Healthcare institutions of different levels (tertiary, secondary, primary, and unrated) were selected based on institutional scale, service capacity, geographical distribution, and research feasibility. Multiple clinical departments covering major specialties were selected from each participating institution as survey sites. Consecutive recruitment was conducted at each site, and participants completed an online questionnaire either on-site via QR code or through online channels. To protect participant privacy and improve response willingness, specific departmental affiliation was not collected in the individual questionnaire. All participants participated voluntarily after being fully informed of the research purpose, data confidentiality rules and their right to withdraw at any time.

### 2.3 Inclusion and exclusion criteria

#### 2.3.1 Inclusion criteria

Eligible participants were classified into three stakeholder groups, each subject to corresponding inclusion criteria. Healthcare professionals were defined as individuals currently engaged in medical or healthcare technical work, including physicians, nurses, and other allied health professionals, with relevant professional knowledge and clinical experience. Patients were individuals receiving medical services at the participating institutions who had normal cognitive and communication functions and were able to complete the questionnaire independently. The other stakeholders group consisted of hospital administrators, logistics staff, family members of patients, and other relevant personnel who had a basic understanding of healthcare services and AI technologies and provided voluntary informed consent to participate.

#### 2.3.2 Exclusion criteria

Participants were excluded from the final analytical sample if they had cognitive impairment or psychiatric conditions that prevented independent completion of the questionnaire, if they were unable to comprehend the core concept of generative AI even after on-site explanation, or if they submitted incomplete questionnaires or showed clear signs of careless responding.

Before the formal survey, a standardized definition of generative AI and clinical application examples were provided to all participants to ensure a consistent conceptual basis for responses. The inclusion criterion of basic understanding was implemented through the informed consent process: participants independently judged whether they could understand the questionnaire content, while investigators provided on-site assistance for basic conceptual questions. No separate forced screening item was set to avoid increasing response burden or introducing additional selection bias.

### 2.4 Sample size calculation

Sample size estimation was performed using an *a priori* power analysis approach for multiple linear regression conducted with G*Power 3.1 (18). A conservative medium effect size of f^2^ = 0.15 was specified based on previous empirical studies of healthcare technology acceptance. With *α* = 0.05, power = 0.80, and three core predictor variables in the main model, the minimum required sample size was 77. Considering invalid sample exclusion and the need for subgroup analyses, the target sample size was set at no less than 500. The final sample of 533 valid questionnaires fully met the statistical requirements of the study.

### 2.5 Research instrument

The study employed a self-developed structured questionnaire, developed following a standard three-phase procedure to ensure content validity and clinical applicability: literature-based item pool construction, expert consultation, and pilot testing (19). First, an initial item pool was generated based on established scales from prior international research on healthcare AI acceptance and ethical perception, with all items adapted and contextualized to the specific scenario of generative AI–assisted medical decision-making. Second, multiple experts specializing in medical ethics, public health methodology, and clinical practice were invited to evaluate the content validity and wording appropriateness of the initial items; items were revised, rephrased, or removed based on their feedback to improve readability and clinical relevance. Third, a small sample of eligible participants was recruited for pilot testing. Following item analysis and preliminary reliability testing, items with poor discrimination or low item-total correlation were further adjusted to form the final version of the instrument. The final questionnaire comprised two sections—a demographic information form and the Cognitive Scale for GAI-Assisted Medical Decision-Making—totaling 29 items, including two ranking questions and one open-ended question.

#### 2.5.1 Demographic information questionnaire

The demographic section collected data on nine characteristics: gender, age group, participant identity, level of healthcare institution, years of professional experience, professional title, educational level, occupation, and history of chronic diseases. Branching logic was applied to ensure targeted item presentation: institution level, years of professional experience, and professional title were presented only to healthcare professionals, whereas occupation and chronic disease history were presented only to patients and other stakeholders.

#### 2.5.2 Cognitive scale for GAI-assisted medical decision-making

All measurement items (see Appendix A) were developed in alignment with the extended Technology Acceptance Model (TAM)-based analytical framework outlined in the Introduction. The four core constructs—perceived functional value, perceived ethical risk, acceptance intention, and ethical governance expectations—are well-established concepts that have been widely validated in existing technology acceptance and AI ethics research (8, 12). Each construct was adapted from previously validated scales with appropriate contextual adjustments for the GAI-assisted medical decision-making scenario and is grounded in corresponding theoretical foundations:

Perceived functional value (4 items) was adapted from the classic perceived usefulness construct of TAM (7), measuring respondents’ perceptions of GAI’s ability to improve diagnostic efficiency, reduce medical errors, support complex case management, and lower healthcare costs.

Perceived ethical risk (4 items) was adapted from established risk perception scales in line with risk perception theory (16), focusing on four core ethical concerns in medical AI applications: data privacy breaches, algorithmic bias, erosion of professional autonomy, and diminished humanistic care in physician–patient interactions.

Acceptance intention (6 items) was adapted from classical technology acceptance scales (15), assessing willingness to adopt GAI-assisted decision-making across multiple dimensions, including confidence in collaborative decision-making, willingness to authorize anonymized medical data use, and acceptance of AI-generated clinical recommendations.

Ethical governance expectations (3 items) were developed based on existing AI governance research consistent with medical ethical governance perspectives (17), measuring respondents’ demand for normative oversight of medical GAI applications, including stricter regulatory access standards, specialized training and certification for clinicians, and mandatory algorithmic interpretability. In this study, ethical governance expectations are hypothesized to positively predict acceptance intention: higher demand for standardized governance reflects recognition of the need for responsible AI development, which in turn strengthens user confidence and willingness to adopt the technology.

All items were rated on a 5-point Likert scale (1 = strongly disagree, 5 = strongly agree), with higher scores indicating stronger endorsement of the respective construct.

### 2.6 Reliability and validity testing

Psychometric properties of the scale were evaluated using SPSS 26.0, including internal consistency reliability, convergent validity, and discriminant validity.

For reliability, Cronbach’s alpha coefficients for the four dimensions ranged from 0.802 to 0.924, and the overall scale Cronbach’s alpha was 0.932, indicating excellent internal consistency. Composite reliability (CR) values for all dimensions ranged from 0.872 to 0.941, all above the conventional threshold of 0.7, further supporting the reliability of the scale.

For validity, the Kaiser–Meyer–Olkin (KMO) value was 0.907 (χ^2^ = 6086.828, df = 136, *p* < 0.001), and Bartlett’s test of sphericity was statistically significant, indicating that the data were suitable for factor analysis. Exploratory factor analysis (EFA) extracted four factors consistent with the theoretical structure, with a cumulative variance explained of 73.616%. All items exhibited factor loadings above 0.60 on their corresponding dimensions. The average variance extracted (AVE) for each dimension ranged from 0.629 to 0.826, all exceeding the 0.5 threshold, demonstrating good convergent validity. Discriminant validity was also supported: the square root of AVE for each dimension was greater than the correlation coefficient between that dimension and any other dimension.

### 2.7 Data collection and quality control

Data were collected via the Wenjuanxing online survey platform and distributed on-site in the form of scannable QR codes across selected departments. Trained investigators instructed participants to complete the questionnaire independently on their own mobile devices. Investigators provided only procedural guidance (e.g., clarifying item wording and navigation) and reminded participants of unanswered items, without inducing or directing response content.

Standardized quality control procedures were implemented throughout the data collection process. All investigators completed standardized training prior to the survey to ensure consistent implementation of the protocol. The survey was conducted anonymously, and participants were fully informed of the study purpose, confidentiality measures, and their right to withdraw at any time to reduce social desirability bias. Following data collection, questionnaires with patterned responses, excessively short completion times, or missing data exceeding 5% of core items were excluded from the final analysis. Valid data were imported into an Excel database and coded for statistical analysis.

It should be noted that the on-site organization and reminder procedures may introduce potential response bias. Although all investigators adhered to a neutral protocol, the presence of research staff may have influenced responses to some extent; this limitation is addressed further in the Discussion.

The questionnaires were distributed on-site via scannable QR codes. With investigators present to assist with operational questions and remind participants of unanswered items, the on-site completion rate was relatively high. After quality screening, questionnaires showing patterned responses or missing data exceeding 5% of core items were excluded. Finally, 533 valid questionnaires were included in the final analysis, corresponding to a valid completion rate of 98.7% among all distributed questionnaires. Notably, this high completion rate is partly attributable to the on-site presence of investigators—the same survey setup that may introduce potential response bias, as noted above and further discussed in the Limitations section.

### 2.8 Statistical analysis

Descriptive statistics were computed for all variables: continuous variables were presented as mean ± standard deviation (x̄ ± s), and categorical variables as frequencies (n) and percentages (%). Normality of continuous variables was assessed using the Shapiro–Wilk test, skewness and kurtosis coefficients, and normal P–P plots of regression residuals. For missing data, complete case analysis was applied, and samples with missing values on core variables were excluded; no data imputation was performed, given the very low proportion of missing values in the dataset.

Pearson correlation analysis was conducted to examine bivariate linear associations among the four cognitive dimensions. One-way analysis of variance (ANOVA) was used to compare cognitive scores across demographic subgroups. For subgroups with a statistically significant overall ANOVA result, Tukey’s HSD *post hoc* test was applied for pairwise comparisons to correct for multiple comparisons and control the family-wise Type I error rate.

Multiple linear regression was performed to identify predictors of acceptance intention, with acceptance intention as the dependent variable and perceived functional value, perceived ethical risk, and ethical governance expectations as independent variables. Multicollinearity was evaluated using the variance inflation factor (VIF), with VIF < 10 indicating no significant multicollinearity.

Notably, although the study employed a cluster-based recruitment approach at the department level, cluster identifiers (e.g., specific department or institution affiliation) were not collected in the individual questionnaire to protect participant privacy and improve response willingness. Accordingly, intra-cluster correlation coefficients were not calculated, and multilevel modeling to adjust for clustering effects was not performed. This methodological limitation is discussed further in the Limitations section.

## 3 Results

### 3.1 Sample characteristics

A total of 533 valid questionnaires were included in the final analysis. Participants were recruited from healthcare institutions of varying levels in Henan and Guangdong provinces, comprising healthcare professionals, patients, and other healthcare stakeholders. The sample exhibited good diversity across demographic characteristics (Table 1).

**Table 1:** Demographic characteristics of the study participants.

Demographic characteristics	Category	Frequency	Percentage (%)
Gender	Male	211	39.6
	Female	322	60.4
Age	18–25 years	202	37.9
	26–35 years	186	34.9
	36–45 years	102	19.1
	46–55 years	31	5.8
	≥56 years	12	2.3
Identity	Healthcare professionals	293	55.0
	Patient	104	19.5
	Others	136	25.5
Medical institution level (*N* = 293)	Tertiary	248	84.6
	Secondary	25	8.5
	Primary	13	4.4
	Unclassified	7	2.4
Years of professional work (*N* = 293)	1–3 years	119	40.6
	4–10 years	74	25.3
	11–20 years	73	24.9
	>20 years	27	9.2
Professional title (*N* = 293)	No title	84	28.7
	Primary title	83	28.3
	Intermediate title	108	36.9
	Associate senior title	13	4.4
	Senior title	5	1.7
Education	Primary school and below	7	1.3
	Junior high school	16	3.0
	High school/technical secondary school	24	4.5
	College	28	5.3
	Bachelor’s degree	330	61.9
	Postgraduate	128	24.0
Occupation (*N* = 240)	Freelance	61	25.4
	Institution staff	52	21.7
	Enterprise staff	44	18.3
	Government staff	5	2.1
	Flexible employment	8	3.3
	Others	70	29.2
History of chronic diseases (*N* = 104)	Yes	41	39.4
	No	58	55.8
	Unclear	5	4.8
Total		533	100.0

The “other healthcare stakeholders” group included hospital administrators, logistics staff, and patient family members, with relatively high internal heterogeneity. This group was used primarily as a reference for descriptive subgroup comparisons, and no in-depth analysis of its internal influencing mechanisms was conducted in this study.

### 3.2 Descriptive statistics of core variables

Normality assessment showed that, given the relatively large sample size, the Shapiro–Wilk test indicated statistically significant deviation from strict normality for all four dimensions (all *p* < 0.001). However, absolute skewness values were below 1 and absolute kurtosis values below 2 for all core variables, falling within the widely accepted range for approximate normality. Additionally, residuals from the multivariable regression model approximately followed a normal distribution, satisfying the assumptions for parametric analysis.

Overall, participants reported a moderate level of acceptance of GAI-assisted medical decision-making, with the highest mean score observed for ethical governance expectations (Table 2).

**Table 2:** Descriptive statistics of core cognitive variables.

Variable name	Sample Size	Minimum	Maximum	Mean	Standard deviation
Perceived functional value	533	1.00	5.00	3.87	0.73
Perceived ethical risk	533	1.00	5.00	3.34	0.77
Acceptance intention	533	1.00	5.00	3.68	0.70
Ethical governance expectations	533	1.00	5.00	4.03	0.76

### 3.3 Correlation analysis among cognitive dimensions

Pearson correlation analysis revealed varying degrees of linear relationships among the four cognitive dimensions (Table 3).

**Table 3:** Pearson correlation coefficients among the four cognitive dimensions.

	Acceptance intention	Perceived functional value	Perceived ethical risk	Ethical governance expectations
Acceptance intention	1			
Perceived functional value	0.703**	1		
Perceived ethical risk	−0.006	0.035	1	
Ethical governance expectations	0.623**	0.452**	0.109*	1

^**^Correlation is significant at the 0.01 level (2-tailed).

^*^Correlation is significant at the 0.05 level (2-tailed).

Key findings are as follows:

(1) Perceived functional value was strongly positively correlated with acceptance intention (r = 0.703, *p* < 0.01).
(2) Ethical governance expectations showed a moderate positive correlation with acceptance intention (r = 0.623, *p* < 0.01).
(3) Perceived ethical risk showed no significant correlation with acceptance intention.
(4) A weak positive correlation was observed between ethical risk perception and ethical governance expectations.

### 3.4 Group differences in cognitive dimensions

One-way analysis of variance (ANOVA) was used to examine differences in cognitive scores across subgroups defined by participant identity, educational level, age, chronic disease status, and healthcare institution level. Tukey’s HSD *post hoc* test was applied for pairwise comparisons to correct for multiple comparisons only when the overall ANOVA reached statistical significance.

#### 3.4.1 Identity differences

For acceptance intention, the overall ANOVA showed a significant difference across the three groups (*F* = 4.39, *p* = 0.013). *Post hoc* tests confirmed that both healthcare professionals (3.73 ± 0.70, adjusted *p* < 0.05) and patients (3.71 ± 0.72, adjusted *p* < 0.05) had significantly higher acceptance intention than the other healthcare stakeholders group (3.55 ± 0.67). No significant difference was found between healthcare professionals and patients (adjusted *p* > 0.05).

Healthcare professionals also scored higher on both perceived functional value (3.95 ± 0.74) and perceived ethical risk (3.31 ± 0.75) than patients (3.89 ± 0.70 and 3.23 ± 0.91, respectively), with the difference in perceived ethical risk reaching statistical significance (*p* < 0.05).

#### 3.4.2 Educational level differences

For ethical governance expectations, the overall ANOVA revealed a significant difference across educational levels (*F* = 11.24, *p* < 0.001). *Post hoc* tests showed that participants with postgraduate education had the highest scores (4.33 ± 0.59), significantly higher than those with a bachelor’s degree (adjusted *p* = 0.019) and those with junior college education or below (adjusted *p* < 0.001).

For acceptance intention, participants with postgraduate education also reported significantly higher scores (3.89 ± 0.63) than those with lower educational attainment (*p* < 0.001), consistent with the pattern that stronger cognitive capacity and ethical awareness are associated with higher acceptance under normative governance.

#### 3.4.3 Age differences

For the two core outcomes (acceptance intention and ethical governance expectations), the overall ANOVA showed no statistically significant differences across age groups (all *p* > 0.05). In accordance with the pre-specified analytical strategy, no further *post hoc* pairwise comparisons were conducted.

At a descriptive level, participants aged 18–25 years tended to report lower perceived functional value (3.73 ± 0.70) and acceptance intention (3.55 ± 0.70) compared with those aged 36 years and above, and ethical governance expectations showed a gradual upward trend with increasing age.

#### 3.4.4 Chronic disease history (patients)

This comparison involved only two groups (patients with vs. without chronic disease), so no *post hoc* correction for multiple comparisons was required.

Patients with chronic diseases reported significantly lower perceived ethical risk (2.96 ± 0.91) than those without chronic diseases (3.43 ± 0.90, *p* < 0.05). The most pronounced difference was observed in perceived data privacy risk (2.71 ± 1.08 vs. 3.57 ± 0.99, *p* < 0.001).

#### 3.4.5 Healthcare institution level (healthcare professionals)

For acceptance intention and ethical governance expectations, the overall ANOVA across institution levels showed no statistically significant differences (all *p* > 0.05). Accordingly, no *post hoc* pairwise comparisons were performed for these core outcomes.

At a descriptive level, healthcare professionals in tertiary hospitals tended to report higher acceptance intention (3.77 ± 0.70) than those in unclassified institutions (3.14 ± 0.63), and primary healthcare professionals showed the highest perceived functional value in managing complex cases (4.04 ± 0.72).

### 3.5 Multiple linear regression analysis of acceptance intention

Multiple linear regression analysis was conducted with acceptance intention toward AI-assisted decision-making as the dependent variable and perceived functional value, perceived ethical risk, and ethical governance expectations as independent variables (Table 4).

**Table 4:** Multiple linear regression analysis of acceptance intention.

Variable	Unstandardized Coefficients		Standardized Coefficients	*t*-value	*p*-value	VIF
	*B*	Std. Error	*β*			
(Constant)	0.466	0.139	–	3.354	0.001	–
Perceived functional value	0.508	0.029	0.528	17.465	0.000	1.257
Perceived ethical risk	−0.061	0.025	−0.067	−2.480	0.013	1.012
Ethical governance expectations	0.360	0.028	0.392	12.882	0.000	1.271
*R*-squared (*R*^2^)	0.615					
Adjusted *R*-squared (Adjusted *R*^2^)	0.613					
Durbin–Watson (D–W) statistic	1.898					
*F*-value (*F* statistic)	282.071					
*p*-value	<0.001					

The model demonstrated good overall fit: the *R*^2^ value was 0.615, the adjusted *R*^2^ was 0.613, and the overall F-statistic was 282.071 (*p* < 0.001). The Durbin–Watson statistic was 1.898, indicating no significant autocorrelation in the residuals. All variance inflation factors were below 2, confirming the absence of substantial multicollinearity among the predictor variables.

Perceived functional value of AI was the strongest positive predictor of acceptance intention (*β* = 0.528, *p* < 0.001), followed by ethical governance expectations, which also exerted a significant positive effect (*β* = 0.392, *p* < 0.001). Perceived ethical risk showed a statistically significant negative association with acceptance intention (*β* = −0.067, *p* = 0.013), but its standardized coefficient was notably smaller than the other two predictors, indicating that its actual magnitude of effect on acceptance intention was limited. This pattern is consistent with the bivariate correlation analysis, which showed no significant zero-order linear association between ethical risk and acceptance intention.

Overall, perceived functional value and ethical governance expectations jointly explained 61.3% of the variance in acceptance intention.

## 4 Discussion

This study analyzed empirical data from 533 participants, including physicians, patients, and other healthcare stakeholders in China. With physicians and patients as the core analytical groups, the study systematically examined acceptance intention toward generative artificial intelligence (GAI)–assisted medical decision-making and its ethical determinants. By integrating technological and ethical perspectives, the findings reveal key determinants of acceptance intention and highlight differences in ethical perceptions across stakeholder groups. Drawing on recent international studies, this section compares the findings with existing literature, discusses their implications in the Chinese healthcare context, and outlines study limitations and future research directions.

### 4.1 Dual technological–ethical drivers of acceptance intention

Multiple linear regression results showed that perceived functional value (*β* = 0.528, *p* < 0.001) and ethical governance expectations (β = 0.392, p < 0.001) were the two dominant positive predictors of acceptance intention, jointly explaining 61.3% of its variance. This finding suggests that GAI acceptance in clinical decision-making follows a dual-drive pattern: technological utility remains the foundational premise, while ethical governance expectations have evolved into a core supporting factor.

From a theoretical perspective, this result extends the classic Technology Acceptance Model (TAM) in the medical GAI context. Consistent with the core logic of TAM, perceived functional value—corresponding to the perceived usefulness dimension—remains the strongest predictor of user acceptance, which supports the cross-context stability of the traditional framework (20). On this basis, this study introduces ethical governance expectations as an extended antecedent variable and identifies its robust positive predictive effect. This suggests that in high-stakes medical scenarios, user acceptance of AI no longer depends solely on technical performance evaluation; recognition of normative governance systems also significantly enhance adoption willingness. This finding expands the explanatory dimension of technology acceptance theory from purely cognitive factors to governance-related factors, and echoes the multinational empirical conclusion of Pham et al. that ethical institutional arrangements are a critical foundation for public trust in medical AI in non-Western contexts (21).

The underlying mechanism for this dual-drive pattern can be further interpreted within the Chinese healthcare context. For healthcare professionals, the functional value of GAI is mainly reflected in reducing the burden of routine work such as medical record writing and literature retrieval, thereby freeing more time for clinical decision-making and physician–patient communication; for patients, it lies in improving the accessibility of medical information and auxiliary diagnostic resources, especially in areas with limited access to high-quality medical resources. Meanwhile, both groups generally recognize that medical AI involves high risks related to life and health, and thus regard standardized supervision as a necessary prerequisite for technology application, which makes ethical governance expectations a key factor second only to functional value.

Notably, perceived ethical risk showed a statistically significant but quantitatively weak negative association with acceptance intention (*β* = −0.067, *p* = 0.013), while its bivariate correlation was not significant. This pattern differs from some Western studies that regard ethical risk as a core resistance factor (22) and constitutes one of the key contextual findings of this study. Two complementary explanations are proposed for this result.

First, the buffering effect of ethical governance expectations weakens the negative effect of risk perception. This study found a weak positive correlation between ethical risk perception and governance expectations (r = 0.109, *p* < 0.05), indicating that users who perceive higher risks do not necessarily reject the technology; instead, they tend to demand stronger regulatory safeguards. When users believe that risks can be effectively controlled through institutional norms, their resistance to the technology will be significantly reduced. This mechanism has also been observed in other developing-country contexts (23).

Second, from a practical perspective, GAI-assisted medical decision-making is still in the early implementation stage in China. Most users have not experienced actual ethical adverse events such as data leakage or algorithmic misdiagnosis, and their risk perception mostly stays at the conceptual level, which has not yet been transformed into strong rejection intention. The public’s overall optimistic attitude toward digital medical technology further weakens the negative effect of abstract risk perception. This finding helps to clarify the boundary conditions of ethical risk in technology acceptance: the inhibitory effect of risk perception may be more significant in scenarios where adverse events have been widely exposed, while in the early stage of technology adoption, its role is often overshadowed by value perception and governance expectations.

### 4.2 Differences in acceptance and ethical perceptions across stakeholder groups

One-way ANOVA showed that age, participant identity, educational level, and chronic disease history were significantly associated with acceptance intention and ethical perceptions of GAI-assisted medical decision-making. These differences likely reflect variations in professional responsibility, cognitive capacity, and priorities across stakeholder groups within the Chinese healthcare system.

Healthcare professionals reported significantly higher ethical risk perception than patients. This may be attributable not only to their stronger professional awareness as clinical decision-makers, but also to the specific occupational environment of Chinese clinicians. Within China’s current healthcare governance system, healthcare professionals bear primary responsibility for clinical outcomes and face stricter professional regulatory requirements and greater medico-legal liability. Algorithmic bias, data breaches, and other ethical issues may directly lead to medical disputes and even disciplinary actions, contributing to clinicians’ heightened awareness of potential risks. This is consistent with the findings of surveys among UK general practitioners that front-line clinicians are more sensitive to ethical risks such as algorithmic bias and erosion of professional autonomy (24).

In contrast, patients showed relatively lower ethical risk perception, especially in terms of data privacy. This cannot be explained solely by information asymmetry regarding AI ethics (25). Within the Chinese healthcare context, patients generally have high trust in public medical institutions (26), and often default that hospitals will properly keep medical data confidential; at the same time, under the background of the national promotion of digital healthcare, the public generally holds an optimistic attitude toward the auxiliary role of new technologies. These factors jointly reduce the sensitivity of patients to abstract ethical risks.

Educational level showed a consistent dual effect: participants with postgraduate education had both the highest ethical governance expectations and the highest acceptance intention. This suggests that stronger cognitive ability and ethical awareness do not necessarily lead to technological resistance; on the contrary, highly educated participants may better appreciate the dual attributes of technology value and potential risks, and thus support the promotion of technology under the framework of standardized governance. This is consistent with the conclusion of Sedaghat et al. that highly educated groups have a deeper understanding of the ethical controversies of medical AI (1).

The age difference also presents a characteristic pattern: participants aged 18–25 had lower acceptance intention than those aged 36 and above, which is contrary to the conventional assumption that young people are more receptive to emerging technologies. One possible explanation is that younger participants with higher digital literacy have more exposure to discussions about ethical risks of generative AI, and thus hold a more cautious and critical attitude (9, 27). These findings suggest that the promotion of medical AI cannot simply rely on the digital dividend of young groups, but needs to respond to their ethical concerns more targeted.

Among patients, those with chronic diseases perceived significantly lower data privacy risks. This is because patients with chronic diseases pay more attention to the practical utility of technology in improving disease management efficiency and long-term health outcomes. When disease management becomes the primary concern, sensitivity to privacy risks may decrease accordingly (28).

### 4.3 Shared patterns and context-specific characteristics

Beyond the heterogeneous perceptions observed across demographic and clinical subgroups, the overall pattern of GAI acceptance identified in this study has broader comparative relevance to the global research landscape.

The findings of this study both align with global research patterns and reflect context-specific characteristics of the Chinese healthcare system, offering empirical insights into GAI adoption in developing countries.

At a general level, the positive association between ethical governance expectations and acceptance intention supports the growing consensus that ethical governance is a prerequisite for the implementation of medical AI. Recent research published in NEJM AI likewise emphasized that robust governance frameworks are essential for the responsible deployment of AI in healthcare (29). In addition, the associations of cognitive capacity and priority needs with ethical perception and acceptance intention are consistent with the international ethical governance framework proposed by Gorelik et al. (6), suggesting that the complementary roles of technological value and ethical safeguards may represent a common pattern in medical AI adoption.

At the contextual level, the ranking analysis showed that both physicians and patients in China tended to identify physicians as the primary responsible actors in GAI-assisted medical decision-making. This finding may reflect a more centralized distribution of medical responsibility shaped by collectivist cultural norms, differing from findings reported in Western studies that emphasize patient autonomy in decision-making (30).

Moreover, physicians in unclassified or primary healthcare institutions demonstrated lower acceptance intention compared with those in tertiary hospitals. This may reflect stronger concerns regarding reliability, interpretability, and ethical governance of GAI technologies. It also highlights disparities in healthcare resources and raises important considerations regarding equity in the implementation of medical AI (31). These findings provide valuable insights for developing countries seeking context-sensitive ethical governance strategies for medical AI (32).

### 4.4 Practical implications for the ethical implementation of GAI-assisted medical decision-making

Based on the core findings of this study, the following targeted strategies are proposed to promote the responsible implementation of GAI-assisted medical decision-making, with each recommendation directly informed by the empirical findings.

First, strengthen the value foundation for technology adoption by prioritizing improvements in clinical utility. Given that perceived functional value is the strongest predictor of acceptance, the primary task of promoting medical GAI is to improve its accuracy and practicability in real clinical scenarios, thereby improving diagnostic efficiency, reducing medical errors and optimizing treatment plans. On this basis, tailored initiatives to improve AI ethics literacy should be implemented: professional training on AI-assisted decision-making ethics is provided for healthcare professionals to clarify decision-making boundaries and responsibility attribution; popular science education on medical AI is carried out for patients to enhance their rational cognition of technology value and potential risks (33).

Second, respond to widespread governance expectations by establishing a comprehensive ethical governance framework across the entire AI lifecycle. Since ethical governance expectations are the second strongest predictor of acceptance, accelerating the construction of medical AI ethical norms and supervision systems is not only an ethical requirement, but also a practical need to promote technology adoption. Drawing on the framework proposed by Pham et al. (21), it is necessary to strengthen algorithm transparency and data security review in the access stage, standardize the use behavior of healthcare professionals through certification systems in the implementation stage, and establish a monitoring and accountability mechanism for ethical incidents in the post-deployment stage, so as to thereby strengthening public confidence in the standardized development of the technology through a complete governance system.

Third, implement targeted strategies tailored to the cognitive characteristics of different stakeholder groups. For patients with chronic diseases who pay more attention to disease management effects, while maximizing the practical value of GAI, data encryption and personalized authorization mechanisms should be used to balance practical functions and privacy protection (34, 35). For primary healthcare institutions with lower acceptance intention, GAI systems should focus on operational simplicity and result interpretability, and strengthen technical and ethical training for grassroots medical staff, thereby helping to narrow the digital divide in medical AI application (36, 37).

Fourth, embed ethical principles into technology design to proactively mitigate potential risks. Although the current inhibitory effect of perceived ethical risk appears limited, potential risks should be prevented from the technical source. Principles such as informed consent, non-maleficence and justice should be integrated throughout the entire research and development process (38, 39): privacy-preserving computation and data anonymization technologies are used to protect patient privacy; diverse training datasets are used to reduce algorithmic bias; and ethical risk warning mechanisms are set up to prevent excessive reliance on AI output and while preserving the central role of clinical judgment (40, 41).

### 4.5 Study limitations

This study has several limitations that should be acknowledged.

First, the sample was recruited from only two provinces in China using a convenience cluster sampling design. Although the sample exhibits good demographic diversity, it cannot be considered fully representative of the national population, and the generalizability of the findings should be interpreted with caution.

Second, the cross-sectional design precludes causal inference regarding the relationships among the study variables. Additionally, cluster identifiers were not retained in the individual dataset to protect participant privacy; therefore, potential clustering effects could not be accounted for, which may have affected the precision of the standard error estimates.

Third, in terms of measurement, detailed process records of questionnaire development (including the exact number of expert consultants and precise pilot sample size) were not systematically archived, limiting full traceability of the instrument development process. Furthermore, no dedicated quantitative item was included to measure participants’ familiarity with generative AI, so the potential moderating effect of prior AI knowledge could not be examined. The on-site data collection procedure, which contributes to the high valid completion rate, may also have introduced mild response bias despite investigators adhering to a neutral protocol, as participants’ responses could have been subtly influenced by the presence of research staff. Additionally, this study only employed exploratory factor analysis (EFA) to examine the factor structure of the adapted scale; confirmatory factor analysis (CFA) with an independent sample was not conducted to further verify the stability of the measurement structure, which represents another limitation of the current instrument validation.

Fourth, the “other stakeholders” group was defined broadly and includes heterogeneous subgroups such as hospital administrators, logistics staff, and patient family members. This group served only as a descriptive reference in the present study, and its internal heterogeneity was not explored further.

Finally, additional variables, such as prior AI use experience and institutional digitalization level, were not included in the analytical framework. Future research should address these limitations through nationwide multicenter studies, longitudinal designs, and more comprehensive measurement of relevant variables.

## 5 Conclusion

This study investigated acceptance toward generative artificial intelligence (GAI)–assisted medical decision-making and its ethical drivers using empirical data from 533 participants, including physicians, patients, and other relevant stakeholders recruited from healthcare institutions at different levels in Henan and Guangdong provinces in China. Using a cross-sectional survey and multivariable statistical analysis, the study identified key determinants of acceptance intention and examined differences in ethical perceptions across stakeholder groups, providing empirical evidence to support the responsible implementation of GAI in healthcare decision-making.

The findings indicate that perceived functional value of GAI and ethical governance expectations are the two strongest positive predictors of acceptance intention, jointly explaining 61.3% of its variance. While technological performance remains the primary determinant, clear ethical governance and regulatory safeguards also play a crucial role in shaping acceptance. Perceived ethical risk showed a statistically significant but quantitatively weak negative association with acceptance intention. One possible explanation is that individuals with greater awareness of ethical risks simultaneously demand more robust ethical governance, and such regulatory expectations may buffer the negative impact of risk perception on adoption willingness.

Significant differences were also observed across stakeholder groups. Physicians demonstrated higher perceived ethical risk than patients, while individuals with higher education showed stronger ethical governance expectations and acceptance intention. Younger participants exhibited relatively lower acceptance intention, possibly due to greater awareness of potential ethical risks associated with emerging technologies. Patients with chronic diseases showed lower perceived privacy risk, likely reflecting the higher priority placed on improving disease management efficiency.

Overall, the results support the growing international consensus that successful adoption of medical AI requires both technological effectiveness and robust ethical governance. At the same time, the findings highlight context-specific characteristics of the Chinese healthcare system, such as the central role attributed to physicians in AI-assisted decision-making and greater concerns about technological fairness among primary healthcare institutions. These findings may provide valuable guidance for the development of ethical governance frameworks and context-sensitive implementation strategies for GAI in healthcare, particularly in developing countries. Although this study provides important empirical evidence, further research involving broader samples and more comprehensive analytical frameworks is needed to deepen our understanding of the factors influencing GAI acceptance in medical decision-making.

### 5.1 Contribution summary

This study makes three incremental contributions to existing literature on medical GAI acceptance by addressing several important gaps in current research.

First, in terms of empirical evidence, this study simultaneously examined the acceptance level and influencing factors of GAI-assisted medical decision-making among Chinese healthcare professionals and patients, and systematically reveals the cognitive heterogeneity between the two core stakeholders. Unlike most previous studies that recruited either clinicians or patients alone, this study provides paired comparative evidence from both key stakeholder groups, thereby complementing the limited availability of comparative evidence in the existing literature. Moreover, by providing empirical evidence from a developing country characterized by uneven healthcare resource distribution and distinctive physician–patient relationships, this study enriches the evidence base currently dominated by studies conducted in Western healthcare systems (25) and offers context-specific evidence for evaluating the applicability of existing technology acceptance theories.

Second, in terms of theoretical expansion, this study incorporates ethical governance expectations into the analytical framework of technology acceptance, and demonstrates its significant positive association with acceptance intention, which thereby extending the classic TAM framework in the medical ethics scenario. The finding that perceived functional value is the core predictor supports the cross-context stability of TAM’s core logic; the finding that ethical risk has a statistically significant but relatively small effect complements the inconsistent conclusions on the role of ethical perception in existing studies, and helps to clarify the boundary conditions of risk perception in technology acceptance. The identification of the positive role of governance expectations expands the analytical perspective of existing acceptance research from pure cognitive factors to governance-related factors, providing a new entry point for subsequent research on AI ethical governance and user acceptance.

Third, in terms of practical implications, this study provides quantitative empirical evidence for the formulation of ethical governance policies for medical GAI in the Chinese context. The identified dual-drive pattern indicates that promoting medical AI requires both technological advancement and institutional development; the identified cognitive differences among groups provide a basis for formulating differentiated promotion strategies. These findings may inform regulatory authorities in developing access standards, support healthcare institutions in designing AI ethics training programs, and assist technology developers in optimizing product design, thereby promoting the responsible and sustainable implementation of GAI in clinical decision-making.

## Data Availability

The original contributions presented in the study are included in the article/supplementary material, further inquiries can be directed to the corresponding authors.

## Appendix A

Core Likert-scale measurement items

All attitudinal items adopt a 5-point Likert scale (1 = strongly disagree, 2 = disagree, 3 = neutral, 4 = agree, 5 = strongly agree). The original survey used interrogative statements, which are retained below without rephrasing to maintain consistency with the distributed questionnaire.

1. Perceived Functional Value (Items 10–13).

10. Do you agree that generative AI can help healthcare professionals improve the efficiency of diagnosis and treatment?

11. Do you agree that generative AI helps reduce accidental errors and human negligence in medical practice?

12. Do you agree that generative AI can handle complex cases and provide solutions that healthcare professionals may overlook?

13. Do you agree that the use of generative AI for auxiliary decision-making helps reduce medical costs in the long run?

2. Perceived Ethical Risk (Items 14–17).

14. Do you agree that the application of generative AI may lead to leakage of patients’ personal medical data?

15. Do you agree that generative AI algorithms may contain bias and result in misdiagnosis or unequal treatment?

16. Do you agree that over-reliance on AI will weaken clinicians’ independent clinical judgment and professional skills?

17. Do you agree that AI intervention will create an impersonal doctor-patient relationship lacking humanistic care?

3. Acceptance Intention (Items 19–24).

19. Compared with decisions made solely by healthcare professionals, do you have higher confidence in the collaborative decision-making mode of healthcare professionals plus generative AI?

20. Would you agree to authorize your anonymized medical data for AI training under data security guarantees to obtain more comprehensive diagnosis suggestions?

21. Would you accept healthcare professionals using generative AI to assist your diagnosis and treatment in future clinical visits?

22. If your attending physician recommends AI auxiliary treatment plans, will you agree to adopt them?

23. Do you think AI auxiliary tools are necessary for health management of common and chronic diseases?

24. Do you think it is necessary to recommend AI-assisted diagnosis and treatment services to your family and friends?

4. Ethical Governance Expectations (Items 25–27).

25. Do you agree that far stricter access and supervision standards should be formulated for medical generative AI compared with ordinary software?

26. Do you agree that clinicians using AI auxiliary decision-making should receive dedicated training and qualification certification?

27. Do you agree that medical AI systems must be able to explain the logical basis of their clinical recommendations?
